# Life experiences after kidney transplantation in adolescents: A qualitative meta-synthesis

**DOI:** 10.1371/journal.pone.0321588

**Published:** 2025-04-09

**Authors:** Wenjuan Zhang, Jiaqi Wang, Ziyu Sun, Yuhong Wu

**Affiliations:** School of Public Health and Nursing, Hangzhou Normal University, Hangzhou, China; Tribhuvan University Teaching Hospital, NEPAL

## Abstract

**Purpose:**

To systematically evaluate the life experience of adolescents after kidney transplantation.

**Methods:**

We use computer search Web of Science, PubMed, Cochrane Library, Embase, EBSCO, CNKI, Wanfang, VIP database, search database to October 2024, screening after kidney transplant adolescents life experience of qualitative study. We used the Australian Joanna Briggs Institute Critical Appraisal Checklist for Qualitative Research to quality evaluation, and using the thematic analysis method to integrate the results.

**Results:**

7 articles were included, and 31 research results were integrated into 8 new categories and combined results are 3 integration results: complex emotional experience; eager for multifaceted support; self-adjustment.

**Conclusion:**

Nursing staff should pay attention to the psychological experience and needs of adolescents, to eliminate their stigma, to promote positive cognition, and to improve their quality of life.

## 1. Introduction

Chronic kidney disease (CKD) is one of the serious health risks for adolescents, with a global prevalence of approximately 8.5%-9.8% [1] and a mortality rate that is 30 times higher than that of normal adolescents [2]. Kidney transplantation is an effective means of improving the prognosis of chronic kidney disease [2], providing adolescents with a life-saving opportunity and restoring their normal studies and daily life to a large extent. However, the benefits are accompanied by various risks. Post-kidney transplant patients need to take immunosuppressive drugs for a long period of time as prescribed by the doctor. Studies have shown that adolescents undergoing kidney transplantation are 2.5 times more likely to be non-compliant than adults, and are more likely to develop acute rejection and complications, significantly shortening the lifespan of the transplanted kidney and the patient [3]. Several studies have shown that the changes in physical appearance brought about by medication after renal transplantation cause minors to develop a sense of stigma [4], often feel anxious, fearful and depressed, have difficulty adapting to daily life, and lose their bright expectations for the future, which leads to a decrease in treatment adherence [5,6]. In addition, the combination of the growth process of minors and the process of adaptation to renal transplantation and the emergence of psychological problems can lead to a crisis of identity self-identity and negatively affect the future growth and development of the patient [7].

Therefore, this study through the integration of adolescents after kidney transplantation life experience of qualitative research, aims to understand adolescents real feelings, existing problems and psychological needs, for clinical kidney transplantation teenagers intervention plan to provide reference, to help adolescents actively debugging, improve the quality of life.

## 2. Methods

### 2.1. Study design

We conducted a qualitative meta-synthesis. This systematic review was undertaken following the Preferred Reporting Items for Systematic Reviews and Meta-Analyses (PRISMA) [8] and the Enhancing Transparency in Reporting the Synthesis of Qualitative Research [9]. The study is already registered at the International prospective register of systematic reviews (PROSPERO), The registration number is the CRD42024574623.

### 2.2. Search strategies

We use the computer search 8 databases (CNKI, VIP, Wanfang, PubMed, Web of Science, Embase, Cochrane Library, EBSCO) on the qualitative experience of adolescents after kidney transplantation, search time limit for self-built library to October 2024, using the combination of mesh heading and free words, retrieval strategy is preset. The search terms included: kidney transplantation, renal transplantation, renal transplantations, transplantations renal, transplantation renal, grafting kidney, kidney grafting; adolescent, child, teens, teenager, youth, juvenile, students, teenagers; qualitative research, ethnographic, phenomenological, grounded, hermeneutics, descriptive, focus group, Interview, content analysis. Finally, 7 qualitative studies on the living experience of adolescents after kidney transplantation were included.

### 2.3. Study selection

Study screening was independently completed and checked by 2 researchers (Zhang and Wang) trained in evidence-based care. The screening of the study was divided into 3 sections. Firstly, All literature were imported into Endnote X9 literature management software for remove duplicate published articles; Secondly, researchers read the title and abstract to exclude the obviously non-conforming literature, and then read the full text to determine the final inclusion. The criteria used to screen the literature for this study were based on the population, phenomena of interest, context, and study design (PICoS) strategy developed. Inclusion criteria: ① population: adolescents after kidney transplantation; ② phenomenon of interest: psychological experience, feeling, needs, response, challenges, etc; ③ context: the daily life scene of the teenagers after kidney transplantation (including school, home, nursery, etc.); ④ study type: qualitative study, including but not limited to phenomenological study, rooted theory, ethnography, ethnography, etc. Exclusion criteria: ① mixed studies cannot extract data from qualitative studies; ② cannot obtain full text or incomplete data; ③ review, conference literature, etc; ④ Non-English literature.

### 2.4. Appraisal of methodological quality

Methodological evaluation of the included literature was evaluated using the JBI Critical Appraisal Tool by 2 researchers (Zhang and Sun). This quality assessment tool consists of 10 questions, each with four answers (yes, no, unclear, not applicable) [10]. Fully meeting the criteria was graded A, partially meeting it was graded B, and not meeting it at all was graded C; literature of grade B and above was included in this study. In case of disagreement between 2 researchers discuss with the 3rd researcher (Wu) to decide whether to include or not.

### 2.5. Data extraction

Two investigators (Zhang and Wang) independently used the JBI Qualitative Assessment and Review Instrument Data Extraction Tools for Qualitative Research (JBI-QARI) checklist [11] to extract data from the qualifying study into a Microsoft Excel spreadsheet, including (i) researchers; (ii) countries; (iii) methods; (iv) populations; (v) themes, participants’ quotes; and conclusions. If there is a difference in perception between the two researchers, the decision is made by the corresponding author with more experience and expertise.

### 2.6. Data synthesis

The Thomas and Harden thematic analysis approach was used to guide the meta-synthesis [12], and the purpose of this analysis was to synthesize the life experiences of adolescents after kidney transplantation, Since the thematic analysis enables the analysis, organization and detailed description of the data, it enables the authors to gain insight into the themes emerging in the data during the reading and analysis of the data [13]. First, the first author constantly read the results and participants’ quotes extracted from the studies and coded them. The corresponding author checked all the original data and coding, and if the opinion occurred, conducted a group discussion to determine the coding situation. After coding all the studies, the first and second authors developed themes. Secondly, the first author constantly induction and summaries by comparing similar and different coding situations to determine themes and subthemes. Finally, the first author read the initial data again to determine that no other new codes and themes appeared, and the corresponding author again checked the themes identified entirely.

### 2.7. Rigor, trustworthiness, and reflexivity

Our study members have a background in nursing and specialized training in evidence-based medicine, and all studies are independently evaluated by two researchers to ensure the credibility of the study; Thematic analysis was used to enable the researcher to gain insight into the content and themes of the study and to analyze the experience of living with a kidney transplant to ensure the reliability of the study; Our findings selected participant quotes to support the study theme, and in the included studies included patients from different countries, ages, and cultures, reflecting the confirmability and transferability of the study. In addition, the researcher was completely faithful to the original patient’s expression during data analysis, without substituting personal feelings and thoughts, which improved the accuracy of the findings.

## 3. Results

### 3.1. Basic characteristic of study

**Fig 1:**
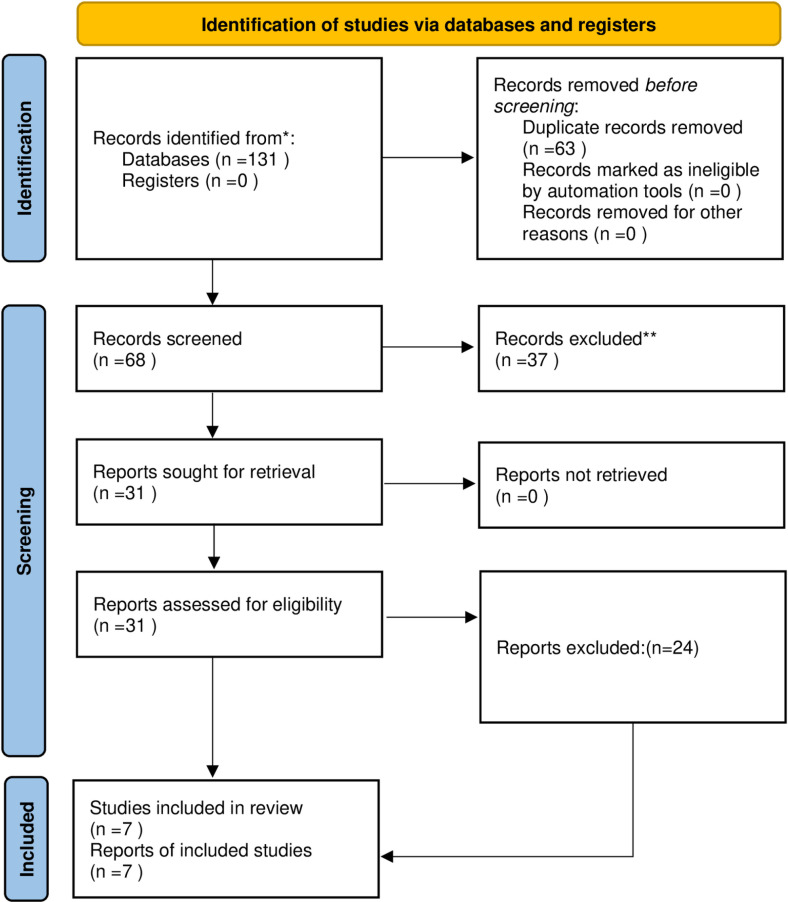
PRISMA flowchart.

We searched 131 studys through 8 databases, removed duplicate studys, after repeated reading, 7 studys were included [14–20], a total of 85 people were included, studies were conducted in Canada (n = 2), New Zealand (n = 1), South Korea (n = 1), United States (n = 2), Australia (n = 1); The 7 studies included used different qualitative research methods, 2 phenomenological studies, 1 descriptive qualitative study, and 4 qualitative studies. The literature screening process is shown in Fig 1 (PRISMA flowchart). Basic characteristics of the included literature are shown in Table 1.

**Table 1:** Included the basic characteristics of the literature.

Study	Country	Research Method	Study Population	Phenomenon	Main Result
Leblond 2020	Canada	phenomenological approach	10 adolescents who had received a kidney transplantation	Describe the experiences of adolescents undergoing kidney transplantation, and the development of their identity.	6 Results: self-concept, body image, social relationships, relationships with parents, career choices, unique experiences of adolescents receiving donations from their parents.
Walker 2019	New Zealand	qualitative research	13 teenagers and adolescents who had received a kidney transplant	Exploring the experiences and expectations of teenagers and adolescents who had undergone kidney transplantation.	3 Results: aiming at transplantation, addressing negative emotions, and enhancing understanding and knowledge.
Kim 2016	Korea	Descriptive qualitative study	9 adolescents who underwent a kidney transplant	Understand the experiences of Korean adolescents undergoing kidney transplant surgery.	6 Results: Different, not being invited as decision makers, being one of them, still being different, having mixed feelings for the mother, and dealing with new situations.
Korus 2011	Canada	phenomenological approach	8 adolescents who underwent a kidney transplant	To explore the needs of adolescents undergoing kidney transplantation.	2 Results: views on transplantation experience and coping with transplant experience.
Dunbar 2022	America	qualitative research	10 adolescents with kidney transplantation and 9 caregivers	Experience of adolescents and teenagers after transplant.	5 Results: Exchange information: Information user and information contributor, management transition: family management and self-management, building confidence: worry and confidence, telling your story: concealment and self-expression, normal kidney transplant: feeling different and feeling the same.
Pollack 2021	America	qualitative research	13 adolescents with kidney transplantation and 11 family caregivers	Understand the experiences and challenges of adolescents transplant recipients and their families.	5 Results: daily barriers, meaning building, transition and agency, social interaction, community engagement and support.
Tong 2011	Australia	qualitative research	22 teenagers and adolescents with kidney transplantation	To explore the experience of teenagers and adolescent kidney transplant recipients after kidney transplantation.	4 Results: obtaining a normal sensation, facilitator, barriers, and information needs.

### 3.2. Quality appraisal

The quality evaluations of the seven included studies were all grade B. The philosophical basis of 7 studies is consistent with methodology; the methodology of 7 studies is consistent with research questions or research objectives; The methodology of 7 studies was consistent with data collection methods; 7 studies were consistent with data representativeness and representativeness and data analysis methods; 7 studies were consistent with results interpretation; 2 studies explained the status of researchers from the perspective of cultural background and values; 1 study addressed the impact of the investigator or the study on the investigator; 7 studies’ subjects and opinions were typical; 6 studies were approved by the ethics committee; and 7 studies were derived from the analysis and interpretation of the data. Methodological Quality appraisal is presented in Table 2.

**Table 2:** Methodological quality appraisal.

Included study	①	②	③	④	⑤	⑥	⑦	⑧	⑨	⑩	Quality Appraisal
Leblond	Y	Y	Y	Y	Y	Y	Y	Y	U	Y	B
Walker	Y	Y	Y	Y	Y	Y	N	Y	Y	Y	B
Kim	Y	Y	Y	Y	Y	N	N	Y	Y	Y	B
Korus	Y	Y	Y	Y	Y	N	N	Y	Y	Y	B
Dunbar	Y	Y	Y	Y	Y	N	N	Y	Y	Y	B
Pollack	Y	Y	Y	Y	Y	N	N	Y	Y	Y	B
Tong	Y	Y	Y	Y	Y	N	N	Y	Y	Y	B

**Legend:** Y = Yes; N = No; U=Unclear; NA=Not applicable.

Adapted from JBI Critical Appraisal Tool (2015), https://jbi.global/critical-appraisal-tools.

Notes: Q1: Is there congruity between the stated philosophical perspective and the research methodology? Q2: Is there congruity between the research methodology and the research question or objectives? Q3: Is there congruity between the research methodology and the methods used to collect data? Q4: Is there congruity between the research methodology and the representation and analysis of data? Q5: Is there congruity between the research methodology and the interpretation of results? Q6: Is there a statement locating the researcher culturally or theoretically? Q7: Is the influence of the researcher on the research, and vice- versa, addressed? Q8: Are participants, and their voices, adequately represented? Q9: Is the research ethical according to current criteria or, for recent studies, and is there evidence of ethical approval by an appropriate body? Q10: Do the conclusions drawn in the research report flow from the analysis, or interpretation, of the data?

### 3.3. Meta-synthesis results

The researchers analyzed the literature, refined 31 research results, summarized 8 new categories, and obtained 3 integrated results comprehensively.

#### 3.3.1. Integration theme 1: Complex emotional experience.

(1) **Self-identity crisis:** Adolescents may feel a gap between themselves and other normal peers. This is manifested in their feelings of vulnerability and weakness during medical treatment. (Hospitalisation and medical procedures constantly remind me that I am a ‘patient’ [14]). The weight gain associated with immunosuppressive drugs is a significant factor in why some people feel more abnormal after transplant than before. As time passes after the transplant, participants begin to realise that they are still different from other people and feel frustrated. They gradually learned that there were still some restrictions on diet, medication and activity. In particular, their dependence on immunosuppressants became a major source of difficulty in post-transplant life, as the medication interfered with their daily (I take my pills at six in the morning. I am not fully awake when I take my pills. Then I slept lightly until seven. As a result, I was exhausted at school. Having to fast for an hour before taking my medication is also frustrating [16]) (My medication makes me feel different because I have to take it to protect my kidneys. Other people just take pills to get rid of colds and stuff like that. I have to take medication to survive. It makes me feel different. I take medication that other people can’t take [18]) (My face swelled up and I gained a lot of weight. I’ve been transplanted for 2 years and I still haven’t grown taller [20]). Some young people felt that their personality and temperament had changed since their transplant. They talked about losing self-esteem, feeling alienated and lonely, and becoming more withdrawn and isolated during the kidney transplant experience. They struggled to get on with good peers and lost confidence (I don’t really know my personality anymore, I used to be shy but now I’m very outgoing. It frustrates me because I find it annoying not to know what kind of person I’m going to be. A lot of people find it a bit weird and hard to adapt to [20]). Physical changes make it difficult for these adolescents to accept their bodies, which greatly affects their self-esteem. They express hatred for their bodies, which leads some to a path of self-harm and low self-esteem (I really don’t like the bloating I get from taking prednisone. I also get teased a lot and it really bothers me [17]). Adolescents reported feeling that they had become a ‘laughing stock’ and that these experiences had left emotional scars (at school, when people made fun of me, I used to walk away and slam into a wall [20]). (I think I would rather die than undergo a medical check-up, be hospitalised or take medication for the rest of my life. Sometimes I think I shouldn’t be saved [16]).
(2) **Anxiety and fear:** They were afraid of returning to their pre-transplant life, with excessive anxiety and fear of an uncertain future (I was depressed after the transplant, I might get cancer or something, I was really worried [15]) (I’m afraid that my kidney won’t last long and I’ll have to have another transplant in a few years [18]). Participants were always ‘afraid of a failed transplant’ and worried that ‘transplants don’t last forever’, which would mean being dependent on dialysis and therapy again. Teenagers said they were very anxious and that their hearts ‘pounded’ when they waited for the results of blood and urine tests for kidney function [17]. In addition, not being invited to be decision-makers before the transplant increased their anxiety after the transplant. Looking back on the day they received their kidney transplant, young people said they were not given enough information about the procedure to express their opinions. They reported being put in a passive position and excluded from important decision-making processes (When the decision was made about the transplant, no one asked me if I wanted it. My parents and health professionals thought I had no idea about transplantation [16]). For teenagers who have received a kidney from a brain-dead donor, the timing of the transplant is always decided suddenly, so that no matter what time, place or activity they are involved in, they have to be rushed to hospital and immediately prepared for the operation. As a result, they feel ‘more confused and scared’ about life after the transplant. (When I got the call, I didn’t know what was going to happen. I was surprised. I immediately turned off my computer and went to the hospital. The kidney had come from a brain-dead donor whom I didn’t know. I was very scared [16]).
(3) **Lack of belonging:** Strong negative emotions, such as shame and the desire to hide scars, prevented them from participating in social activities, which created an impression of isolation and not belonging (I really don’t like myself, I can’t accept my body. It’s the scars that I don’t like. I think it’s ugly. I don’t let myself go swimming with my friends. I really don’t like to talk about my story, and if they see my scars, they ask more questions. I think it’s like a vicious circle [14]) (after the transplant, I feel more disabled and I tend to stay away from people more [15]). Some even mentioned that they wanted to start a new life in a place where no one knew them (I want to be in a place where no one knows I have kidney disease, where I can start a new life as a chef, with well-functioning kidneys [16]). Peer misunderstanding of the disease is another barrier to maintaining intimacy (In gym class, I gave water to a girl who complained of thirst. But she refused to take it because she was afraid my disease would spread through the water [14]) (Oh, you don’t look like me, so why are you near me? Or ‘Why are you with me?’ [18]) (I remember being called a coyote in fourth grade, which made it harder for me to trust people because I was always surrounded by the whole bullying thing. I lost a lot of friends and I was afraid to make friends because I was afraid of getting hurt [20]).
(4) **Perception is overprotected:** All the young people were able to recognise that they were overprotected and some said that their parents had become more anxious and preoccupied after the transplant (When it’s summer I don’t like to wear a mask because it’s too hot. When Mum and Dad are away, I sometimes go out without my mask. If they find out, I’ll be in big trouble. Mum scolds me for going out without a mask [17]). Adolescents who received kidneys from their mothers saw their mothers as saviours who had ‘given them two lives’ [16]. Such adolescents often obey their mothers; in fact, they often feel compelled to obey their mothers, even when they do not want their mothers to control their lives or be overprotective. There are also teens who struggle with getting too much attention at school (I got more attention than the other kids.... But I really don’t like it...... I want to be like everyone else, a normal kid, I want to be treated as an equal [17]) (I don’t need to be watched all the time. That’s one thing I hate about school, they always feel the need to observe me personally. I just hate that and they don’t seem to understand that [19]).

#### 3.3.2. Integration theme 2: Eager for multifaceted support.

(5) **Professional support**: Older teenagers were more willing to know more in order to understand their treatment, and some older teenagers said they felt unprepared as the education and information they received centred on the transplant process and did not include living with a kidney transplant [15]. The vast majority of adolescents say they want to know everything about their disease and treatment [17]. They want to know (a) potential complications; (b) side effects of medications and procedures; (c) how to maintain a healthy lifestyle; (d) expected outcomes for transplant recipients; (e) predictions for the future and implications for school, work, and family; and (f) information about transitioning to adult health services (Doctors and nurses talk about everything, but they mention drugs and alcohol very little. They need to talk more about the effects of alcohol. I don’t drink a lot. But what if I get older and drink heavily? What would that do to me? Then there are drugs, which are illegal and you can’t take them, but thousands of people do. What would happen if I decided to do that? [20]). These adolescents also described receiving professional information in a way that they wanted to look at photos of other adolescents undergoing transplantation, watch video clips of transplantation procedures, or hear about complications experienced by other patients [20]. There were also adolescents who felt that if the information was provided through a website or other computer-based instructional programme, then they could choose a medium that matched their personal learning style [20].
(6) **Develop meaningful social support:** Teenagers having understanding, supportive and caring family members can boost their self-confidence, positive coping behaviours and social adjustment (My parents always told me, don’t worry, it’s not your fault, which was reassuring. The influence of my parents definitely played an important role. I felt bad at first, but they made me feel good [20]). Communication between patients can help teenagers gain experience and help (what helped me the most was going to kidney camps and meeting other people like me, you hear what they’re going through, what they’ve been through and see how they’re coping. Even though the doctors have explained everything, when you meet someone who has been through it makes you worry a little less [20]) (older people who have had a transplant will help you understand what you are going to go through [20]). Being around other transplant patients helps develop and build confidence (I just felt...... I could fit in [18]) (Camp is a really cool place. It’s a place for people with health issues. It’s a place where everyone can be themselves, not be judged, and can be totally accepted [18]). The perception of friends is crucial to a teenager’s self-esteem (my friend told me the other day: I think you’re healthy because you’re healthy and you’re always there (at school), not lying around the house [14]) (I’m now surrounded by people who were good friends a long time ago. And my boyfriend. They are all very supportive [17]). These supports provide encouragement and the development of self-worth for adolescents [17].

#### 3.3.3. Integration theme 3: Self-adjustment.

(7) **Cognitive adjustment:** Over time, teenagers mature and see kidney transplantation positively (I am proud of these scars, I think they look great, I am proud of them [18]) (I will die, but fortunately, because my father gave me his kidney, I’m still alive [18]), the teenagers thinks that receiving the transplant is the beginning of exploring the unknown world, and no longer pays attention to other people’s strange eyes (I really don’t care what other people think of me [18]), start to rethink the meaning of “being different” (I am different, but in a sense, everyone is different, not because of the kidney transplant, but my strengths, weaknesses and others are different [14]).
(8) **Behavioral adjustment:** Teenagers struggle to live after the transplant and begin to share their disease with others (I won’t hide it (scar), they are my friends, they don’t think of me differently [18]), Start to develop interests (he (a friend) is really good, enhanced my confidence in music, we play music together [18]), try to participate in various activities (I would dance, assemble robots, collect items, or trim my nails and chat with friends [16]). In addition, the teenagers will also make a good future career planning (I plan to pursue a business degree next year, hoping to work for an accounting company[20]). (I want to go to a film university[14]).

## 4. Discussion

### 4.1. Pay attention to the negative experience of teenagers and improve their psychological defense ability

First, this study and previous studies show that [21], after receiving kidney transplantation, teenagers will face a series of challenges. Including the fear of rejection, disease complications, Therefore, medical staff should use appropriate communication skills, pay more attention to the non-verbal communication of teenagers, for younger teenagers, auxiliary tools such as drawing and toys can be used to explore the real feelings of teenagers, and personalized intervention guidance for stressors. Secondly, the disease and body appearance changes make teenagers to be ridiculed, strong stigma, self-injury or suicide behavior, so that teenagers are forced to reduce social activities, therefore, hospitals, schools, social organizations should pay attention to them, speeding up the construction of hospital-school linkage management services, through health education, media reports to increase the publicity of basic knowledge of disease, make the public especially teenagers with students correct understanding of kidney disease, to reduce the fear and prejudice of kidney transplant patients [22]. In addition, medical staff should also be able to start from the perspective of humanistic ethics, using situational simulation method, role playing method, stimulate everyone’s empathy, can really understand teenagers, and encourage teenagers to seek help from others, increase teenagers’s psychological defense ability. Finally, the teenagers developed anxiety because the parents’ excessive involvement in disease management affected their autonomy, Therefore, nursing staff should give family members scientific and correct guidance. Some studies have indicated that [23], empowerment support can improve self-care capacity in kidney transplant patients, We can guide the family members to give more affirmation to the teenagers, and gradually transfer the responsibility of disease management to the teenagers, so as to enhance the ability of the teenagers to deal with the problems while avoiding aggravating their rebellious psychology.

### 4.2. Provide multi-directional support to meet the needs of the teenagers

This study found that the teenagers after kidney transplantation are eager to get the support in various aspects, and the positive and effective social support can help the teenagers to better cope with the difficulties in learning and daily life. First of all, teenagers think that family and friends are the closest people, and their understanding, support and encouragement are the catalyst for teenagers to actively deal with the disease. Nursing staff should guide their families and friends to use the Internet to accompany teenagers, increase the sense of belonging, and provide the greatest spiritual comfort for teenagers. Secondly, peer support is more likely to make teenagers have emotional resonance, and group psychological counseling can effectively reduce the negative emotions of patients, improve the quality of life [24], medical staff should make full use of this feature, hold more patient exchanges, summer camp and other collective activities, to carry out multi-person psychological counseling. Thirdly, the teenagers expressed the urgent need for disease-related information. Therefore, medical staff should improve the existing health education content according to this, fully evaluate the disease knowledge needs of the teenagers, and use learning classes and related APPs to push regularly. Finally, the teenagers believe that they do not have enough participation in disease decision-making. Some studies have found that if the teenagers are not fully prepared before transplantation, they lack confidence in the disease management [25]. Therefore, before transplantation, the medical staff should fully communicate with the teenagers, introduce the preoperative and postoperative process to the teenagers, listen to the opinions and suggestions of the teenagers, and respect the decisions of the teenagers.

### 4.3. Stimulate positive emotions and promote self-adjustment in teenagers

The results of this Meta integration show that teenagers will actively adjust themselves as their body and mind become increasingly mature. Teenagers gradually take the disease experience as the beginning of a new life, re-examine the significance of kidney transplantation, personal cognition is the basis of behavioral change, therefore, medical staff should give positive guidance in the appropriate age of teenagers. From the perspective of positive psychology, mindfulness stress reduction, mind thinking, cognitive behavior therapy and so on should be adopted to strengthen positive cognition and help teenagers find self-value. In addition, the teenagers will also take measures to adapt to the post-transplant life, such as expressing themselves, participating in social communication, cultivating interests and hobbies, Bandura’s social cognitive theory [26] suggests that human learning is an observational imitation of others and their reinforcing outcomes, therefore, nursing staff should encourage caregivers to lead by example, and nurses, as medical workers in close contact with teenagers, should also set a good example for teenagers and maintain teenagers’s positive coping behavior.

## 5. Advantages and limitations

To our knowledge, the present study is the first to integrate the living experiences of adolescents after kidney transplantation, in the process of integrating the results, the patients’ own thoughts were preferred, ensuring the accuracy of the research results as much as possible. These studies represent adolescents from different national and cultural backgrounds, providing a richer and more transferable description of the experiences of this population. Limitations include the following points: ① We only included and analyzed the published literature, while those grey literature were not included, this leads to findings that may not be comprehensive; ② It is very pity that the original studies included in this study did not separate the dilemma faced of adolescent patients with different demographic characteristics.

## 6. Conclusion

This study integrates the qualitative study of the life experience of adolescents after kidney transplantation, and systematically and comprehensively interprets the true feelings of teenagers at this stage. Patients undergoing kidney transplant have a psychological and social burden, suffer from physical and mental development, and yearn for social support, but over time, some patients will experience positive adjustment and adaptation. Therefore, medical staff should pay close attention to the negative experience of patients in time, providing support, and guide the development of patients’ positive emotions, so as to help teenagers to achieve positive changes as soon as possible. Meanwhile, future studies should conduct more high-quality studies on adolescents with different demographic characteristics.

## Supporting information

S1 File Dataset. (DOC)

S2 File Literature inclusion and exclusion. (XLSX)

S3 File Search strategy. (DOCX)
