# Supplementary material for: Life experiences after kidney transplantation in adolescents: A qualitative meta-synthesis
Source: PLoS One. 2025 Apr 9;20(4):e0321588. doi: 10.1371/journal.pone.0321588 (PMC11981148; doi:10.1371/journal.pone.0321588)
Supplement: S1 File — (DOC) [file pone.0321588.s001.doc]

**1.Becoming unique: A qualitative study of identity development of adolescent kidney recipients**

Aims of the study: our interest was to describe the experiences of adolescents who have received kidney transplants and the process of identity development in them.

Research method: semi-structured + qualitative study

Study population: 10 adolescents who had received kidney transplants

Research themes: six main themes: self-concept, body image, social relationships, relationships with parents and career choices, unique aspects of the experiences of adolescents who have received donor parents

Data extraction：In October, 2024

1. self-concept: ‘I own a part of someone else; I'm not different, I'm different’

Self-concept is a prominent theme that we present first because it is at the centre of adolescent discourse. Adolescents struggle between feeling normal and being different. Symptom presentation, physical appearance, maturity and acceptance of illness, as well as peer acceptance all move them towards or away from feeling normal. Adolescents are ambivalent when dealing with the concepts of normal and abnormal. When they angrily compare themselves to others, they define themselves as ‘normal,’ a sense of ordinariness that stems from the perceived similarity between their own concerns and those of their friends (e.g., school, graduation, finding a job, and participating in social activities). However, they felt alone in other ways, and this sense of a gap between them and other teens was distressing. A sense of leading a dysfunctional life was associated with several aspects of their experience. Firstly, for many, the vulnerability and debilitation they felt during medical complications made them feel abnormal, rather, the number of hospitalisations and medical procedures reminded them that they were a ‘patient’. (Symptomatology) The moment they were transplanted took on different meanings for different adolescents, for some of them it was the beginning of what they perceived to be a more normal life, and after the transplant life became more about exploration and self-fulfilment than about ‘surviving’ and coping with constant medical appointments, and thus became a catalyst for identity development. catalyst for identity development. However, others had a different experience and believed that transplantation would lead to disability. These young people belonged to a minority group who saw themselves as sick, abnormal and more vulnerable than their friends. They wish they could enjoy the same lifestyle and freedom as everyone else. Weight gain from immunosuppressive drugs is one aspect (physical appearance) that causes some to feel more abnormal after transplantation than before. Secondly, maturity and acceptance of their health status affected how they viewed themselves. Adolescents who showed more maturity, and who had accepted to some extent a condition that needed to be managed in the long term, saw themselves as healthy but different from the norm, with values passed on to them by their parents such as acceptance and love of themselves making them feel normal, and for some being different was described as a very normalising and positive thing. They shared that everyone is different in some way and that this way of thinking implies resilience. They saw themselves as different not because of transplantation but because of their values, personality and temperament (maturity and acceptance of illness). ----- ‘I am different, but in a sense everyone is different. Yes, I had a kidney transplant, but it wasn't because the transplant made me feel different. I feel different for a lot of reasons, my strengths, my weaknesses, but it's not because I have a kidney, everyone is different’. Thirdly, peer perceptions are crucial to adolescents' self-esteem. Most people do not want to feel exposed or weak in front of others, especially in front of teens their own age. Many expressed a strong desire to be seen as someone who is different, rather than being called a patient. Those who felt accepted by their peers felt more normal than those who experienced rejection; in short, seeing themselves as a patient, coupled with feelings of abnormality, and having to cope with peer rejection appeared to serve as an experience in their endeavour to build a positive outlook and reach a realistic identity. -- ‘My friend told me the other day: ’I think you're healthy because you're healthy and you're always there, not like you're lying at home, sick.’ It got me thinking, and I've talked to my mum about it. Yes, I've had a transplant and there's a lot of side effects and I'm tired ‘’ if someone was looking at me, he wouldn't know I've had a kidney transplant. So, I don't think I'm a sick person.’

2. Body imagery: ‘My body is the sanctuary of my journey’

Boys and girls were equally concerned about body image, which was a major theme in their conversations, and which was influenced by illness and received body markers. Scars and other physical characteristics such as looking younger or being overweight, often associated with substance use, were the most commonly cited early symptoms for these adolescents, and changes in their bodies made them feel vulnerable and exposed because it was a visible manifestation of their illnesses and a reminder that they were sick. For some, strong negative emotions, such as shame and the desire to hide scars, prevented them from participating in social activities. This created an impression of isolation and not belonging. Physical alterations made it difficult for these young people to accept their bodies, which greatly affected their self-esteem. Most of them harboured preconceived ideas about their body image and aspired to change their bodies in order to look more like their peers. Many adolescents, mostly girls, emphasised the importance of accepting their bodies as they are because it represents their journey and therefore their strength and resilience. Accepting one's body was seen as a long process, sometimes exacerbated by concerns about body signs (e.g., scars, being overweight). People's responses to body image ranged from pride and not wanting to hide their scars and trying to own their story, to expressing self-hatred about their bodies, which led some people down a path of self-harm and low self-esteem. Older adolescents seem to be more accepting of their bodies than younger people, but this is not always the case. Emotional responses to body changes resulting from medical procedures therefore need to be considered in the process of identity development, and scars can prevent some adolescents from engaging in the social interactions that are necessary to form an identity, leading to feelings of isolation. ----- ‘I really don't like myself, internally or externally, but I think I can accept my illness. I don't accept my body. What I don't like are the scars. I think it's ugly. I don't let myself go swimming with my friends. They can tell me they think it's beautiful, but I really don't like it. But I think there's some kind of connection between the two ...... I'm very uncomfortable talking about my story, what I'm going through because of my physical condition. If they saw my scars they would ask more questions. I think it's like a vicious circle.’

3. Social sphere: ‘My friends, they accept the real me.

The social domain was also present in their discourse, focusing on acceptance, the hardships of creating a sense of belonging, and the importance of trust in fostering openness. Adolescents felt that it was powerful and satisfying to talk about their medical problems in relationships where they could truly be themselves. Most of them had good relationships with their peers and felt accepted in their circle of friends. However, sometimes feeling more mature than adolescents their own age can present difficulties in choosing friendships and drive them to isolate themselves from others. In their self-definition, their survival rate due to illness and treatment was seen as positive, but the thought of a gap between them and their peers was felt negative. They often felt frustrated at not being able to do the same activities or engage in certain behaviours that would make them feel part of a group, such as drinking. For some, missing days of school can lead to rejection and intimidation when they return to school, making it harder for them to feel appreciated and accepted by a group of friends. A lack of belonging and a lack of shared interests can be considered a challenge to identity development. ----- ‘They are completely different, I'm not very close to anyone, like I have one closest best friend. I think a lot of people think I'm close to them, but I'm not. Like our parents and stuff like that because, it's hard for me to share that with other people and it creates some barriers between me and other people, I don't know, maybe it's just people I know, maybe it's just my age, I don't know, maybe it's because of people I know, maybe it's because of my age but I find it hard for people to acknowledge or talk about it and I find it really embarrassing that they don't acknowledge it. It can create some barriers.’ All of the adolescents talked about their health condition more with old friends than with people they knew. They felt they couldn't talk about their condition with people they had just met because the disease represented a secret garden. The teenage girls had more open conversations with their friends about their health condition and they felt more accepted. Social relationships were positive and contributed to a sense of belonging. One teen emphasised the importance of having friends with good health habits. When asked about romantic relationships, the importance of being accepted and being willing to talk about their health were important factors in their willingness to be in a relationship. Some felt they were not ready, while others felt that past relationships had not lasted. Some felt they were not ready, others felt that past romantic relationships had not lasted. In these teens, transplant surgery did not necessarily seem to be a barrier to a relationship. Some felt they weren't ready, others felt that past romantic relationships hadn't lasted. In these teens' eyes, transplant surgery doesn't have to be a barrier to a relationship. ---- ‘My last relationship was okay, it went well. We told each other everything and we trusted each other. We broke up because of distance, but the transplant doesn't stop me from having a boyfriend or girlfriend like everyone else. I talk about it all the time and they'll say ‘well, I didn't even notice’. That's just it, it's not a barrier to a relationship.

4. The family sphere

The family, and more specifically the relationship between adolescents and their parents, was another important theme. Participants talked about intimacy, communication, overprotection, and the need to prove to their parents that they are no longer vulnerable, but strong.

4.1 Intimacy and Communication Adolescent families differed in terms of intimacy and communication. Almost all adolescents described their relationship with their parents as good. The most appreciated element of this relationship is an open communication and the feeling of being supported in the face of difficulties and daily struggles related to the physical condition. Communication based primarily on therapy and daily life can lead to a less satisfying relationship, with the belief that their parents will always be there to support them, making them feel safe and more willing to explore their environment. Exploration is part of active behaviour and is essential in shaping one's identity. --- ‘My parents and I are both very close and very open, we talk about everything and I don't hide anything from them. We have a great relationship. I also feel closer since the transplant. It's weird. We're definitely better than we were and we understand each other.’

4.2 Feeling overprotective All of the young people were able to recognise that they developed being overprotected, with some young people saying that their parents were always overprotective, possibly due to their health condition, whilst others said that their parents had become more anxious and preoccupied after the transplant. Overprotection was a significant barrier to their desire to be more autonomous, and they felt that the attitudes of their overprotective parents prevented them from reaching important milestones in adolescence, such as autonomy, independence and exploration. Some feared that they would have to do more and more things on their own. Some of them still relied on their parents for basic daily chores, such as cleaning the house, while others were more autonomous. Overprotection is somehow thought to contribute to a sense of security within the family, whereas the idea of living without the constant presence of such a safety net can cause distress. Thus, overprotection, when too intense and bossy, can lead to adolescents being afraid of exploring and engaging in interests outside of the home and developing an identity that will not step outside of their comfort zone. --- ‘They are strict, but in a good sense. Sometimes we think they're too strict, but it's in a good sense; when I was little, my mum was overprotective. She had a lot on her plate; she stopped working. Because I was overprotected as a child, I got into a lot of trouble with childcare. I didn't want to leave home.’

4.3 I'm stronger than you think When asked how they thought their parents viewed them, most of the adolescents thought their parents thought they were brave, strong and mature. However, a few felt neither trusting nor strong in the eyes of their parents, and the majority of adolescents were able to understand how transplantation had impacted on the lives of their parents, both donor and non-donor, and hoped that their parents would let go of the experience of illness in order to support their growing independence. From the adolescents' perspective, letting go of the disease was difficult for their parents, but necessary for their development. --- ‘Last year, I was at a party at the end of the school year. Some boys came and built a camp fire. When my friends left, I stayed with them. But now my mum doesn't want me to go because she says they're not my age, but most of them are 14 - 15; it's boring because that's not how I make new friends. My mum has had her ups and downs because of my transplant. I have a friend who smokes and she thinks I'm impressionable, but I don't care at all.’

5. Vocational/professional: ‘Questions about the future’

For many, transplantation is a catalyst for identity development as it leads to teens having more energy to explore, leading to a sense of having new possibilities, however, for some it is still seen as a barrier to exploring and engaging with desirable interests in professional, athletic, and personal realms. Adolescents must find a compromise between what they want to do and what they can do. Many are eager to explore different areas of interest and possible future careers, but few actually engage in activities related to these interests. Some described a tendency to put off exploration and engagement because they experienced limitations when they were sick (e.g., months in the hospital, home dialysis, weeks in bed) and showed little motivation. Despite having to cope with limitations, most adolescents embraced a new value:giving to others. They expressed a desire to pursue a career in the health sciences. Such a programme uses their experience in a positive way to help others.

6 Unique aspects of the experience of adolescents who receive donor parents

6.1 From Receiving to Owning Receiving parental donation seemed to have a positive impact on self-definition, mainly because of the way these adolescents perceived their parents' attitude towards donation. They felt that their parents were very supportive of the idea that this kidney was now theirs, which gave them a sense of normalcy and allowed them to integrate this new organ as their own. Adolescents described the close relationship they had with their parents. After donation, their relationship became deeper and they felt closer to their parents than they had ever felt before with their donation

6.2 Debt or gratitude? The experience of receiving a donation from a parent was painfully affected when teenagers thought about performing acts that could potentially put corruption at risk. Some express guilt about the donor parent's involvement in these behaviours. Others had different experiences, with teenagers mentioning how careful they were with their new kidney and the importance of looking after it well, as it was a gift they had received. They did not necessarily feel indebted to their parents. In both cases, the grafts from their parents were very important gifts to be cherished, but the adolescents‘ experiences differed in terms of their sense of responsibility for their parents’ donation. In this sense, some people's guilt can inhibit exploration and thus hinder identity development. ---- ‘It's like in the bank, you can pay it off, but a debt like this, you can never pay it off, if you make a mistake, if you don't take your medication, for example, she gave me a second life. I always felt like I owed her something. I always felt I had to think about the future, not make mistakes, not lose my graft. I think about the future not the present, a little bit.’

6.3 What if I need another kidney? Some teenagers who had received a donor organ from their parents thought in advance that they would need another organ later. They asked themselves who would be the donor and who would help them. These participants felt anxiety and distress when they thought that their parent donor would one day no longer be there to help them. -- ‘I'm grateful because I tell myself that when I'm old, I mean, I don't know, maybe I won't have a son like that, a son who would do everything he could to help me, who would donate a kidney to me. In fact, it scares me a little bit. I saw an old man on Facebook walking down the street holding a sign that said he was looking for a kidney for his wife. I don't want to be on dialysis when I grow up and do nothing.’

**2.Children's experiences and expectations of kidney transplantation: A qualitative interview study**

Aims of the study: we explored the experiences and expectations of children and young people who have undergone a kidney transplant.

Research method: semi-structured interviews Qualitative study

Study population: 13 children

Study themes: aiming for a transplant, dealing with negative emotions, improving understanding and knowledge

Data extraction：In October, 2024

1. Aiming for transplantation

The only real treatment Children report hearing from clinicians and their families that kidney transplantation is their best treatment option, especially superior to dialysis. Children can articulate the expected benefits of transplantation:------ ‘’It will make me feel better, it will make me feel stronger‘’ Most children see transplantation as the only treatment option to improve their quality of life. Often children did not mention the possible side effects of the unfavourable conditions of transplantation, instead focusing on the fact that transplantation was a positive outcome and a solution to their health problems. ----- ‘I felt like I had a problem and it was finally going to be solved. When I finally knew I was going to be transplanted and everything was going to be okay, it was a really good feeling.’

Avoiding dialysis All of the children who had received dialysis treatment talked about their intensely negative experiences and aversion to the treatment. ----- ‘’I would 100% rather have a transplant than dialysis. After the transplant, everything went back to normal.’ They described the ‘exhaustion’ they felt during dialysis because of the restrictions it placed on their normal activities. Even children who had gone through turbulent times after receiving a transplant still saw it as a way of escaping the burden and difficulties of dialysis:---- ‘I remember feeling very restricted - my freedom and some of my independence; I felt like it had all been taken away from me. Restrictions of being physically connected to machines every night, not eating certain foods, not participating in certain activities’

2. dealing with negative emotions

Coping with anxiety and fear Children spoke openly and clearly about how kidney disease and transplantation had affected their emotions and coping, with children describing that they were aware of how anxiety, fear and sometimes depression had affected their lives. For some, this anxiety was linked to the possibility of not being able to find a donor and receive a kidney transplant and the fear of acute complications during the transplant process ----- ‘’I remember being really worried and remember a lot of anxiety and fear about not being able to find a kidney donor."’ ‘‘I was worried that something bad would happen during the transplant.’’ Some of the older children openly discussed in detail the lack of support they had received in dealing with their emotions during the transplant process and post-transplant clinical care. ---- ‘I don't remember anyone telling me then or now (how I coped) anything like that.’

For some children, this fear and anxiety was constant and related to their fear that the transplant could fail at any time, - she said:- ‘I don't like the fact that I'm in danger of it dying again, and then I've got to have the operation, and then we're going to have to do it, so that's something I really don't like. I don't like to risk it dying’ or that their transplant medication might have side effects ----- ’I was so depressed after the transplant that I might get cancer or something and I was really worried’

Guilt towards siblings Children talked about how the transplant had a negative impact on their relationships with their siblings, made worse by the geographical distance between the family and the transplant centre, where they would need to live for a long period of time for transplant tests, surgery, post-operative care and for complications to arise. ----- ‘When you're away from them, especially when you're sick, you realise how much you want them and how much you really need them’ Children lose the security and familiarity of being with their siblings, but they also feel guilty knowing that their siblings don't get the attention of, amongst others, their carers. attention from others.

The burden of parents as donors Children described the complex emotions and intense anxiety of having a parent donate a kidney, and for many they expected or hoped that their parent would be with them in their care, but unexpectedly their parent was being cared for in another (on the same campus) adult hospital. For many of the children, who were very familiar with the children's ward, they described how they had seen donors receiving far less favourable care, which added to the stress and uncertainty, with children worrying about not knowing if their parent donors were ok, and feeling guilty that their parents' care was perceived to be less favourable than their own. ‘I was going to throw up when I went to see him because I was in my room with my mum and then I went to see him and I thought I was going to be with him and he was with me and he deserved better care than me.’ One child said that she would have done better with the transplant if her parents had had more support; she said, ‘I think there must have been nurses, they must have comforted the child and I think the parents definitely needed more comfort.’

3. Improving understanding and knowledge

Personalised information Children expressed expectations and preferences for information about transplantation, depending on their age, with younger children preferring reassurance and older children and adolescents wanting to know about the process and treatment. Prior to undergoing the transplant procedure, many of the younger children expressed a desire not to know too much, which they explicitly described as a way of managing their anxiety. At this stage of the transplant process, they needed reassurance rather than information. -- ‘I think they told Dad most of it when they had the transplant, and that's okay because I don't really need to know at all.’ Older children were more likely to want to know more in order to understand their treatment and to be more actively involved in their care, and some older children said they felt unprepared for the fact that the education and information they had received had focussed on the transplant process and had not included life with a kidney transplant. --- ‘I didn't really know what to expect’ Many children described the potential of using technology and animation as a way of learning to help with understanding, perseverance and self-management. Some had initiated this themselves, searching websites for age-appropriate educational information or using ‘fitbit’ and mobile phones to receive information and reminders that were more socially acceptable, particularly amongst young people. -On days when I'm drinking (and taking pills) I'll set an alarm for half an hour and my mum usually does it for me and then we'll have a drink every half an hour so it's a lot easier, it's really simple and it means that my mum doesn't have to nag me anymore.’ It's also a cool thing to do in the fashion world, my friends love it, my friends just keep pressing the button, it's so cool

1. reassurance from peer support Those children who were able to talk to other children in similar situations gained encouragement by talking to others who had been through the same process. They felt reassured when they heard that their peers were feeling well and having positive experiences after the transplant. Peer relationships also provided emotional support. One child described her experience with others and how she saw the benefits of doing this:---- ‘’They found it really helpful and they were all asking me, is it scary?’ I could tell them, but I was telling them that different people might feel differently as well, and if I did, it would be great.’

**3.Experiences of Korean adolescent renal transplant recipients**

Aim of the study: to understand the experiences of Korean adolescents who have undergone kidney transplantation.

Research method: Descriptive qualitative research Semi-structured interviews + Focus group interviews

Study population:9 adolescents who received kidney transplants

Research themes: being different, not being invited as a decision maker, being one of them, still being different, having complicated feelings for their mothers, coping with new situations

Data extraction：In October, 2024

1. Being different All adolescents undergo haemodialysis or peritoneal dialysis to maintain their kidney function until a kidney transplant. Teens report that they often feel different from their friends and that their bodies ‘look like robots’. The medication and dialysis equipment installed resulted in limited movement and changes in appearance, and the teens said that before the transplant they felt like a ‘block of wood’ because they lived with too many limitations and needed to remain passive in many physical activities. One adolescent said, ‘While on peritoneal dialysis, I was often hospitalised for peritonitis. When I first started dialysis, I was a boy with a big belly. I had a hard time making and keeping friends’ (Participant 1). In particular, adolescents reported feeling like they had become ‘the butt of the joke,’ isolated from their peers because of their appearance (e.g., ‘short and hairy’), and that they were often bullied by their peers and referred to as ‘monsters’ or ‘hairy’. monsters’ or “monkeys”. These experiences leave emotional scars.

2. Not being invited as decision makers Looking back to the day they received their kidney transplant, the adolescents said they were not given enough information about the procedure to be able to express their opinions. They reported that they were put in a passive position and excluded from key decision-making processes: ‘When the transplant decision was made, no one asked me if I wanted it. My parents and medical staff thought I had no idea about transplantation’ (Participant 3). For those adolescents who received a kidney from a brain-dead donor, the timing of the transplant was always decided suddenly, so no matter what time, place, or activity they were attending, they had to be rushed to the hospital and immediately prepared for surgery. As a result, they feel ‘more confused and scared’. ----- As soon as I got the call, I didn't know what was going to happen next. I was surprised. I immediately switched off my computer and went to the hospital. The kidney came from a brain-dead organ donor I didn't know. On the way to the hospital, I kept thinking about the sandwich I forgot on the table that I hadn't finished yet. (Participant 8)

3. Become one of them After the transplant, adolescents initially experience a sense of freedom and feel ‘good and relaxed’. Prior to the transplant, dialysis had severely limited their activities, forcing them to maintain a strict diet and creating some negative emotions. After the transplant, they felt as if they ‘could fly in the sky’ and ‘their stomachs opened up with a bang’ as they were free of the ‘bulky dialysis catheter’. Physical changes such as ‘free urine,’ ‘no swelling in their milky face,’ and ‘no more hairiness’ led them to believe they were becoming normal. They begin to enjoy a ‘normal life’ with fewer challenges and the freedom to spend time with friends. They felt they were going from ‘a different person’ to ‘a normal person’. One adolescent girl said: ‘I don't need regular dialysis anymore. I don't have to be late or miss school’ (Participant 4). Another adolescent said, ‘Now I can play rough with my friends. I also perform martial arts like Hapkido or Taekwondo. I can wear what I want to wear’ (Participant 2). During dialysis, I could not be submerged in water. After the transplant, I went to a water park and it felt great because I no longer had to put bandages on my body and I could spend time with my friends and be no different from anyone else. (Participant 3) Becoming ‘just like any other kid’ and having ‘a life just like any other person’ brought joy and excitement to their lives.

4. Still different As time passed after the transplant, participants began to realise that they were still different from other people and were disappointed. They thought the transplant would make them completely healthy and remove all the limitations in their lives. However, they gradually learnt that there were still some restrictions on diet, medication and activity, and that health management was still required. In particular, their dependence on immunosuppressants became a major source of difficulty in their post-transplant lives, as the medication interfered with their daily lives, activities and peer relationships:I take my medication at six in the morning. When I take my medication, I am not fully awake. Then I sleep lightly until seven. As a result, I am exhausted at school. It was also frustrating to have to fast for an hour before taking my medication. (Participant 8) As a result, adolescents found it difficult to be accepted by their peers due to the fact that they were still different in appearance and needed to follow a strict regimen of immunosuppressant medication, realising that they were ‘still not catching up with their peers in terms of height’ and were still different from others in many ways. Misunderstanding of the disease by their peers was another barrier to maintaining close peer relationships. One adolescent said: ‘In gym class, I gave water to a girl who complained of thirst. But she refused to take it because she was afraid that my disease would spread through water’ (Participant 5).

5. have complex feelings for their mothers During the interviews, adolescents often mentioned their mothers, whether they were kidney donors or not, and they expressed complex feelings for their mothers. Although they gained more freedom after the transplant, they still felt anxious about their overprotective parents. Their mothers often told them to be careful. These mothers were worried and tended to over-parent their children, and many even tried to plan for their children's future by saying, ‘You're not healthy, don't get married.’ One adolescent said : ‘I still wear a mask. She told me that when I need help I have to look sick or weak because I am not a normal person. I'm not well’ (Participant 6). Another adolescent said:I still can't understand my mum, she said that riding the waterslide would cause the transplanted kidney to fall out. I still can't believe it. Although the stitches have been absorbed since the transplant, I think my mum is overreacting. Also, she seems to know it won't happen. However, she seemed worried about the slightest chance. (Participant 5) In order to satisfy their own needs without disappointing their mothers, they sometimes did things secretly:In summer, I don't like to wear a mask because it's too hot. When my mum and dad are not around, I sometimes go out without a mask. If they find out, I'm in big trouble. Mum scolds me for going out without a mask. (Participant 1) Despite the children's discomfort with their mothers' protection, anxiety, and control, they still expressed gratitude to their mothers. In particular, adolescents who received kidneys from their mothers saw their mothers as saviours who had ‘given them two lives’. Such adolescents often obey their mothers; in fact, they often feel compelled to obey their mothers, even when they do not want their mothers to control their lives or be overprotective. These complex emotions often lead to conflicts in their quest for autonomy.

6. Coping with the new situation The majority of adolescents (n = 8) responded with acceptance, optimism and a more active lifestyle regarding the changes and new limitations after transplantation. Being on lifelong medication was their ‘inevitable fate’ and they tried to look at the situation in a ‘positive light’. In addition, to alleviate the associated stress, they tried to participate in various activities and hobbies, such as chatting with friends, dancing, assembling robots, collecting objects, or trimming their nails. In addition, despite their limitations and health conditions, adolescents continued to pursue independence and wanted to be self-reliant when they grew up: ‘I don't want to be dependent on my parents when I grow up. I want to do what I want to do, support my parents and have my own house to live in. But I don't know if that's possible’ (Participant 4). On the other hand, even after a successful transplant, participants always had ‘the fear of an unsuccessful transplant’ and worried that ‘the transplant won't last forever’, which would mean going back to being dependent on dialysis and therapy. Teenagers said they were so anxious that their hearts ‘pounded’ as they waited for the results of blood and urine tests on kidney function. They were afraid of going back to their pre-operative lives and anxious about an uncertain future. Because the transplanted kidney is not working well, my face sometimes turns dark... My friends ask me why my skin has turned dark again. I am still sensitive about my appearance. (Participant 9) I am afraid of having a baby. I don't think I can take care of the baby. If I can't keep myself healthy, how am I going to take care of someone else? (Participant 5)

As a result of this fear and anxiety, they sometimes expressed resentment and complained about their situation: ‘I think I'd rather die than undergo a medical check-up, be hospitalised or take medication for the rest of my life. Sometimes [I think] I shouldn't be saved’ (Participant 8). Other challenges these adolescents faced in coping with life after surgery were hiding their health condition while wearing a mask for protection. They were afraid of drawing attention to themselves, being a burden to others, and being rejected by their peers. ---- ‘When I take my medication, some ignorant friends may ask me why I do it. Then I roughly say it's because I'm sick. I just didn't mention the transplant because it was so humiliating. Explaining the whole story in detail distracts me. It's better when others don't care. Some passed, others asked curiously. I ended up explaining it briefly.’ (Participant 3), some even mentioned wanting to start a new life in a place where no one knew them: ‘I want to start my new life in a place where no one knows that I had kidney disease and received dialysis.’ There, I can start a new life as a chef with a well-functioning kidney. (Participant 6).

4.Exploring the information needs of adolescents and their parents throughout the kidney transplant continuum

Aim of the study: to explore the needs of adolescents who have received a kidney transplant,.

Research method: Phenomenological study Focus group interviews

Study population: 8 adolescents who received kidney transplants

Research themes: perceptions of the transplant experience, coping with the transplant experience

Data extraction：In October, 2024

1. Perceptions of the Transplant Experience Adolescents described the transplant process as very stressful and articulated four major sub-themes of stressors. Adolescents also articulated that they learnt to cope with these stressors by learning about the transplant process and developing meaningful social support. The transplant process is stressful for all adolescents. In particular, this stress was related to body image, wanting to be normal, physical pain and discomfort, and breakdowns in communication with the medical team. Each of these sub-themes is described here with quotes.

Body Image These adolescents expressed how much they hated the adverse effects of medication on their appearance. These changes not only affected their self-image and self-confidence, but also the way others treated them. Teenagers provided examples of how they had been teased or ostracised because of the way they looked. Some described how they lost weight quickly by going on a fast diet. The following example illustrates how two teens feel about their body image because of their medications. ‘I really don't like the bloating I get from taking prednisone. I also get teased a lot and it really bothers me’ (Patient 5, 17 years old). Another teenager said, ‘I'm fat too - I look like a chipmunk.’ Everyone calls me ‘chipmunk’ and I yell back! (Patient 15, 17 years old)

Wanting to be normal Although some of the stressors were related to body image pressures, as described earlier; the pressure to be normal went beyond body image and appearance. Adolescents described the stress of not being able to participate in normal life. For example, they wanted to be able to go to school and spend time with friends. They described pressures related to privilege or treatment that made them feel different from their classmates, and they wanted reassurance that they could live a normal life despite everything they had to go through. The following example illustrates how an adolescent feels when he wants his teacher to treat him like any other student without a chronic illness: ‘I get more attention than the other kids . . . . But I really don't like it ...... I want to be treated like everyone else, a normal kid, I want to be treated as an equal’ (Patient #15, 17 years old). Another described the impact of her different appearance on her peers' acceptance of her: ’Oh well, you don't look like me, you don't look like the popular kids. They're like, ‘Oh, you don't look like me, so why are you near me?’ Or ‘Why are you with me?’’ (Patient 12,17). -May be due to changes in physical appearance

Pain and Discomfort Adolescents described the stress associated with pain and discomfort from surgery, the recovery process, and various procedures such as biopsies and the initiation of intravenous catheters. This stress was primarily experienced at the time of transplantation or shortly thereafter. For example, two teens said, ‘It was just painful . . . . (Patient 4, 16 years old) and ‘When you're in there you have to have a million little tubes and stuff and I can't move’ (Patient 14, 14 years old).

Miscommunication Adolescents described the stress of being unprepared for surgery or complications and not being listened to by the health care team. Adolescents described this stress as a result of not being told what to expect or because what they were told to expect did not happen in the way it was explained. One teenager typified this situation by saying:- ‘I don't think they prepared me enough. They said it wouldn't hurt, but it did. They said I would get knocked out, but I didn't’ (Patient 5, 17 years old). Regarding not being listened to and having to have a biopsy, the adolescent said, ‘I wasn't happy with the process because I felt like they jumped to conclusions too soon.’

2. Coping with the transplant experience Adolescents articulated two primary ways in which they cope with the stress of the transplant experience: (1) gaining knowledge and (2) developing meaningful social support. Access to knowledge was further broken down into 2 sub-themes: (1) the content of the information they found appropriate and (2) the process: how they wanted to receive the information.

Access to Information Participants clearly expressed their interest in gaining more knowledge about the transplant experience. Most indicated that they learned about the transplant experience primarily from their parents and members of their health care team. Overall, they understood and recognised the importance of developing knowledge about their illness and transplant experience in order to become more independent and advocate for themselves. In order to do this, they needed specific information and advice on how to provide this information. (1) Content: the vast majority of adolescents said they wanted to know everything they could about their illness and treatment. They wanted to know (a) potential complications; (b) side effects of medications and procedures; (c) how to maintain a healthy lifestyle; (d) expected outcomes for transplant recipients; (e) projections for the future and impact on school, work, and family; and (f) information about transitioning to adult health services. This need for information was exemplified by one patient who said:- ‘All possible outcomes, like all possible things that could happen, not just some’ (Patient 7, 14 years old). Another adolescent commented, ‘I think the side effects of medication and how they make your body change is a very serious thing’ (Patient 5,17 years old). They wanted some reassurance that their diet and physical activity were positively contributing to their health. They also felt that it was important for others who were about to undergo transplantation to understand the serious consequences of non-compliance with the regime of prescribed medication and other treatments, and how non-compliance could negatively impact on their health outcomes. For example, 1 adolescent said it was important to ‘tell them what happens if you don't take your medication’ (patient 7,14). They also wanted to hear stories of hope that they could live a ‘normal life’ despite all the things they needed to learn and do to keep their transplanted kidney healthy. One teenager, when asked what was the most important thing he wanted to tell another teenager before he received his transplant, said, ‘Just listen to music, talk to your friends, hang out, live your life the way you're supposed to live it, and don't let it, even if you get a kidney transplant, don't let it get in the way of you’ (patients 15,17). (2) Process: these adolescents described a variety of ways in which they wanted to be educated about the transplant experience, and the overriding theme was the variety of options available to them to gain this knowledge. For example, they felt that some teens would want to see pictures of other teens who had received transplants, watch video clips of transplants, and hear about complications experienced by other patients, while other teens did not want these things. As one teen said, I think it should be up to you to decide if you want to see it or not. Yes, if you want to see the footage, you want to face the facts, right? You can ask to see it, and if you don't, they'll tell you (Patient #12, 17 years old). These adolescents felt that if the information was provided through a web site or other computer-based instructional program, then they could choose the medium that fit their individual learning style. They felt that a web-based programme was well suited to tailoring the information. For example, it could give children a tour of the hospital, a step-by-step look at what happens at each stage of the transplant process, introduce members of the transplant team, and allow children to hear real-life stories from other transplant recipients. However, it excludes one-on-one time with members of the transplant team, which teens do not want, as they find that helpful as well. The second major component of this subtheme is that adolescents want to take in information gradually. They do not want to be overwhelmed with too much information at once. As one teenager said:- ‘I'd rather come back to it than have it thrown at me and go crazy from thinking about it too much ...... not being able to deal with it’ (Patient 4, 15 years old). Another adolescent stated that children receive transplants at a very young age and that it is important that as they get older they should learn about their experiences and become more aware of their condition and experiences. He said:If they are very young, like when I was 5, or when they were 10. He should know what he is going through. So, they should tell you slowly, little by little, what happened, why you were in the hospital, why it happened (patient 15,17).

Developing meaningful social support. These adolescents said that developing meaningful social support helped them cope with transplantation. They described two main types of social support:instrumental and emotional. Instrumental:Adolescents described the need to access material or tangible resources. The supports they described included using the Ronald McDonald house to live and go to school, MSN (Microsoft Network), and their parents. One adolescent described how her parents played a key supportive role: ‘My parents helped me a lot in the beginning of medication just to remind me’ (Patient 7, 14), and another described how his grandmother helped him cope with dietary restrictions: ‘Due to the dietary restrictions, my dad's twin brother had it when he was 5 or 6 years old, so that helped me. That's where my grandma and him grew up; they knew what to avoid and stuff like that’ Emotional:These supports provide encouragement and the development of self-worth for adolescents. They were supported by friends, sometimes parents, and adolescent medical doctors. One adolescent said, ‘Now I'm surrounded by good friends from a long time ago. And my boyfriend. They are all very supportive’ (Patient 5, 17 years old). Another adolescent described:I basically forgot everything. Like right now, I'm just, living my own life. Like waking up in the morning, going to school, then coming home and sitting around. On the weekends, hang out with my friends (Patient #7, 14 years old).

5.My Kidney Identity: Contextualizing pediatric patients and their families kidney transplant journeys

Aims of the study: the aim of this study was to explore the information needed to help paediatric patients and their families understand their post-transplant experience

Research method: qualitative research semi-structured interviews

Study population: 10 paediatric kidney transplant recipients and 9 of their primary carers

Research themes: exchanging information: information users versus information contributors, managing transitions: family management versus self-management, building self-confidence: worry versus self-confidence, telling one's story: hiding versus self-expression, normal kidney transplantation: feeling different versus feeling similar

Data extraction：In October, 2024

1 Exchanging Information:Information Users and Information Contributors .

Shifting

In addition, age and experience influence information exchange behaviour and needs. As paediatric patients mature and learn more about their clinical condition, they become information seekers and providers, just like their carers ---- ‘They're part of it, and looking at the whole picture and seeing how much joy they create, what kind of life they create, that's what I want to give back. ’ That's why I want to practice alternative medicine, because it's the doctors. It's the doctors, it's the love I feel from them, it's the connection I have with them. It completely shapes my life."’

Informant

‘I went for three whole years and I was able to talk to the young people there who received kidney transplants. I was able to teach them in my own way. I also looked up at people older than me and asked them to tell me what they were going through’ “I might tell them how I got mine out and how they could get theirs out if they didn't like it.”’ And, of course, I tell them how much work I had to do to get there.’ Patients use their post-transplant experience to support other patients undergoing similar treatments. Several participants commented on the value of attending a camp for paediatric patients with multiple chronic conditions to share their experiences and hear the experiences of other transplant patients.

2. Managing transitions:family management versus self-management Throughout the kidney transplant process, paediatric patients need different types and levels of support from their primary carers to stay healthy and keep their care on track.

Parental management

Not only did our patients describe the support they received from their parents or carers (T4, T14), : ‘Most of the time my dad always tells me what my medication is or when I go to my mum's house they always tell me across the room, “Hey, it's time to take your medication.” ’ Because, well, I was dying, but luckily, because Dad gave me his kidney. I'm still alive. ......’

Parental Management to Self-Management

As patients became more familiar with the tasks required to maintain their health, they transitioned from relying on their families to becoming more self-sufficient. Patients and carers describe how young transplant patients are taking on more responsibility for managing their day-to-day and complex needs, she says: ‘They have always been supportive and helpful, so now I'm just starting to take the first steps in my own life.

Self-management

These self-management tasks ranged from simple tasks such as setting a medication reminder phone alarm, to more complex tasks such as setting a goal ‘T11: ’You set a clear goal and it was clear and you worked towards it and you achieved it ...... So you really learnt how to look after yourself to the point where we don't need it because you can look after yourself without it.’

As expected, the transition to self-management coincided with important developmental and social milestones in patients' daily lives, particularly when adolescents recognised the impact of their kidney transplant on activities such as going to school, playing sport, going to parties or attending concerts T13.1: ’Really, just make sure I bring my medication. Even if I'm just going out for a little while, just to hear someone say, hey, I want to go out more. So, I bring my medication with me. I bring water.’

3. Building Confidence: Worry and Confidence

Worry.

Fear of transplant failure often comes with any new health problem T14: ‘Because I'm worried that my kidney won't last long and I'll have to have another transplant in a few years.’ T7: ‘It's a pre-surgery form and I sometimes get scared before an operation.’

Worry to confidence

Community support from other transplant patients played a key role in helping patients deal with new fears or complications T3: ‘It made me more confident ...... Just talking to people about being ill ...... If I tell them, they'll understand.’ At the time I didn't think anyone would understand me but I know people do if you tell them.’ T9: ‘So from the very beginning I had the confidence and the feeling like any other child who receives a kidney transplant. So that connection that started this whole thing, I needed to share this experience. I couldn't keep this to myself because that would darken my life in a way, ‘Oh, I have to worry about this. I have to worry about this. I can't do that. I can't do that.' I have to share that.’

There was confidence:

As our participants gained more self-confidence, previous stress- and anxiety-stimulating items (e.g., transplanting scars) led to feelings of empowerment (T5).T5: I'm proud to have these scars. I think they look great and I'm proud of them. I've been through a lot. I think my scars speak for themselves. I feel empowered when I have these scars. When I look at my scars, I feel like I can take on the whole world. I feel strong. I feel proud and happy.’ Reporter: ‘How do you feel when you sing that song?’ T15: ‘I felt strong.’

4. Telling one's own story:Hiding and self-expression

An important part of discovering one's current or future identity during the teenage years is learning how to express oneself by telling personal stories.

Hiding one's story

Patients described that in their pre-adolescent to teenage years it was difficult to make trade-offs between who they wanted to be and the expectations or assumptions of others T5:People will put you in a group but won't always react positively. Sometimes you really have to act like you're not (just), especially in high school. Like, I had a lot of friends. I'm kind of cool, and I'm friends with big people. You really have to act like everyone else, especially in high school, or you'll be alienated. You can't be yourself all the time in high school. You have to be consistent with everyone else.’

Individuals also indicated that the complexity of the disease added to the difficulty of telling their story, the extent to which their disease defined their story, and determining with whom to share their story (T7).T7: Because not a lot of people have urinary catheters. And, as I recall you said last time, you don't like people to know you have one, do you?’ ‘Uh-huh ......’ (affirmative).

Patients discussed feeling the need to hide their illness based on previous negative experiences and interactions. Additionally, physical sensations, such as pain, interfered with our participants' desire to share their stories with others (T13.1).T13.1: ’After the transplant, I felt more disabled because of the pain of the kidney transplant, and I was more worried. I tend to stay away from people more.’

Transformation

However, as patients progressed through the kidney transplant process, many began to feel more confident in expressing themselves and communicating the details of their transplant story with others T3: ‘Over time, I seemed to ignore it. If I'm different, I really don't care what people think of me. I still don't care if people think I'm different. Over time you feel different, get over it. ‘When I had my kidney transplant, I felt terrible because my body wasn't normal. I got over it.’

Individuals became more willing to share physical aspects of their illness, such as letting others see their scars or kidney protection (T13.2) T13.2: ‘I think at some point I just stopped caring. Like I wouldn't hide in the bathroom and put it on and tuck it in. I would just say, ‘You know, they're my friends, they're not going to see me any differently.’’

Expression

When patients were unable to express themselves and tell their story through words, they used other methods such as playing the guitar (T5). Finally, patients stated the importance of having a community of supportive people who not only understood what they were going through, but also helped them to self-express (T5).T5: ‘Camp is a really cool place. It's a place for people with health issues. It's just a teen retreat week and everybody has those issues. It's a place where everyone can be themselves, not be judged, and be totally accepted. Where everyone can be confident , where you can be completely yourself. Everything will be fine. This guy, he's been playing for a long time (playing guitar) and he's really good. He really boosted my confidence in music. He said I've been playing for nine months, and I've been playing for nine months, and he said, ‘You're a really good artist. I think you can do something. You'll be somewhere.' And I said, ‘Wow. That's amazing. ‘He's crazy. He plays the sax really well. We just played music together. It was really good.’

5. Normal Kidney Transplant:Feeling Different and Feeling Similar

Feeling different

At different stages of the transplant process, participants discussed how they struggled to find their ‘normal’ After a kidney transplant, people have to do a lot of things every day in order to stay healthy, which further exacerbates the feeling that they are different from their peers who don't have chronic illnesses; participants described having to take medication every day to save their lives , whereas their peers may only need to take medication when they have a cold (T3).T3: ‘My medicines make me feel different because I have to take them to protect my kidneys. There are other people, they just take medication to get rid of colds and stuff. I have to take medicine to survive. It makes me feel different. I take medicine that other people can't take. That's what I think. ...... Taking medicine makes me feel different.’

Participants described their insecurities about the daily demands of physical limitations and illness, which only reinforced their fears when others brought up or pointed out their differences from their peers (T3).T3: ‘I feel like different people might treat you differently. They might treat you in a special way but ...... you just want to be treated like everyone else ...... you shouldn't be treated differently because you have kidney disease. You shouldn't really, but when it comes to things like sports, sometimes you have to. Not all the time ...... it's a really bad situation.’

Transformation

T3 described the importance of eating whatever they wanted after kidney transplantation, as they were no longer subject to the strict dietary restrictions imposed during dialysis

As participants grew older and matured, they stopped worrying about what others thought of them (T13.2) T13.2: ‘...from puberty to maturity I kind of grew up, yeah that happened but I'm still me, I'm not that different from other people.

Same

Finally, finding community with other transplant recipients helped to foster and build confidence in their identity (T11).T11: ‘I was in a place with a lot of people just like them. I just felt ...... I could fit in.’

Whilst kidney transplants can have a lasting impact on the lives of patients and their families, as they grow accustomed to the patient's situation they begin to develop a new ‘normal’ T15: ‘I'm not just a kid who's had a kidney transplant. I can act like a normal kid too.’

**6.****Reflecting on patient-generated photographs of the pediatric renal transplant experience**

Aims of the study: the experiences of paediatric transplant recipients and their family pairs and the challenges they face after transplantation

Research method: Qualitative research Semi-structured interviews

Study population: 13 paediatric kidney transplant recipients and 11 of their parents

Research themes: everyday barriers, meaning-making, transition and agency, social interaction, community involvement and support

Data extraction：In October, 2024

1. Everyday Barriers

Access to Medications Given the rigorous treatment regimen required for kidney transplant recipients, some of the most frequently discussed daily barriers involve medications, especially having the right medication when needed and remembering to take it For students, their schedules for taking medications may conflict with school and extracurricular activities. This forces patients to make more important choices, recognising that their decisions have social, academic and medical consequences. ‘I'd rather miss school than not take my medication because medicine is what really affects me, if I miss school I can talk to my teacher but if I miss my medication it's the other way round’ - t4

2. meaning construction

Fear and uncertainty In everyday life, patients and families must make sense of diagnoses, procedures and outcomes in the context of daily life, family values and available resources (Table 3). However, due to the relative rarity of kidney transplants, transplant recipients and their families often lack peer guidance on how to cope with the challenges associated with transplantation For example, when talking about trying to find the right information, one patient used the visual metaphor of a dim light to explain uncertainty and isolation T4: ‘The lights went out (Figure 2A) because it was like there was nothing, there was no one to really guide you to. There's nothing, like when I was at school, if you had a kidney transplant there's nothing to guide you.’

The New Normal For some younger patients, it was challenging just to understand the meaning of daily life and stick with it, knowing the consequences of failure.T8 ‘Yes, it was a growth and learning experience where I really learnt well, how rare is that? Where did it come from? And what does it feel like for others who have it? It's pretty rare. Just learning about some of the potential symptoms and some of the things that I might have because I have it, it's a lot to learn about, especially about my genetic disease, and I really enjoyed learning about it in science.’

Over time, as patients and families become more experienced, they become more comfortable with uncertainty. When asked how he deals with the fear of upcoming surgery, T7, one of our younger participants said, ‘I've had a lot of surgeries and I've been through a lot, so it's okay.’ For better or worse, these life experiences often help patients with their symptoms, and for many of our participants, meaning-making not only marked the beginning of their transplant journey, but also helped them define normal life after kidney transplantation, including setting realistic expectations for the future. In fact, as our participants became more confident in their abilities, they even recognised their own expertise, which they saw as an opportunity to help others. Our participants wanted to offer a wide variety of advice to their peers, including how to deal with upcoming surgeries, taking medications, or even just learning how to talk to friends about their experiences. When experiences are shared, it's an opportunity to develop empathy and gratitude. ‘When I first started doing it I was much more stressed out but now I feel like, every day when I finish my work and get ready for bed, I go downstairs, go into my room and get everything ready and I think it's necessary and you shouldn't be afraid. I mean you should be more or less prepared T6’

3. Transition and agency

Transfer of responsibility Paediatric patients will ultimately be responsible for their own care, however, the relationship between children and adult carers remains complex as family roles and responsibilities continue to evolve. Some parents of transplant participants noted that their children had not yet developed high levels of self-efficacy or agency. This perception often led to substantial concerns about whether their child was adequately prepared to manage themselves in the future. ‘Because I'm not sick anymore. I can do things on my own, I don't need to be watched all the time. That's one thing I hated about school, having the help of teachers in some of my lessons, they always felt the need to observe me personally. I just hated that. It bothers me because I can do things on my own and they don't seem to understand that.’ T3

Assessing Adherence For some participants, the results of monthly visits to the doctor and lab tests validated the implementation of their treatment plan, and some described a sense of guilt and a decrease in self-efficacy when the results showed negative, unexpected outcomes, and while many adult caregivers described the day-to-day concerns of adhering to their child's care for a long period of time, parents such as P3 realised that, despite the needing to keep a close eye on her child in the past, her child had become more responsible over the past few months. For some carers, it was particularly challenging to recognise a young person's increasing maturity and independence after a long period of heightened scrutiny and close monitoring. This transition is often accompanied by periods of uncertainty, and adults give young people the opportunity to try (and sometimes fail) to adhere to medical procedures on their own. Instead of seeking help from clinicians to improve self-efficacy, reasons for failure to adhere are not fully shared during office visits when feelings of shame and failure are prevalent. ‘I would beat myself up about it. Or my mum wasn't, she was the best mum in the world, but my mum would say, ‘You need to do better.’ I know that's not a good thing to say, but sometimes I feel like I need to say it to myself. You feel like you're the reason for having all this. I didn't look after my kidneys, I didn't do this right, I didn't try’ T5

4. social interaction, community involvement and support At certain times, kidney disease, transplantation and recovery occupied the daily lives of patients, carers and close family and friends, and many participants discussed the value of the physical and emotional support provided by family members to help overcome these challenges. For example, parents described that they placed their child's medication at the prominent centre of the home, promoting shared responsibility and accountability throughout the family, however, our participants recognised that the impact of receiving a transplant is not limited to the patient, but also affects their parents, siblings, extended family, as well as their friends and community. Even our youngest participants feel guilty knowing that their siblings are receiving less attention or missing out on important social events because of transplant-related emergencies or planned medical needs. ‘Without my family, I think I'd be lonely. I get a little tired sometimes of them always reminding me of things, but I know they only do it because they want me to know it's important and they want to remind me how important it all is. It feels so good to have everyone's support.’ T3 ‘When I'm in hospital I don't get to see my sister very much...she spends the least amount of time with her parents. I feel kind of sad that she didn't really get to see me when I was little. [She] felt very left out.’ T14

Many participants talked about how social engagement made them feel supported from the community, and times when they felt excluded or vulnerable due to their illness. For example, a pair of siblings in our participant group (T13.1 and T13.2), both of whom had had kidney transplants, gave very different accounts of the role that family and peer groups played in providing social support.’ It's just, you know, I'm surrounded by people who are too gentle to know what to say or what to do. I can tell, I mean, I can tell when someone is acting differently towards me. ...... I have siblings and very, very close family friends. It's like they're my second parents and they're very supportive. So it's not that I don't feel supported, it's that I feel so supported that it's nobody's fault, if that makes sense. It was just my reaction at the time. And that's how I grew up. I was thinking about my place in the world, my position, who I am- t13.2’

Another patient described feeling frustrated at being alienated due to being treated differently, and at the same time, close family and friends made me feel a little suffocated .

**7.‘‘When I had my transplant, I became normal.’Adolescent perspectives on life after kidney transplantation**

Aims of the study: to explore the experiences and perspectives of adolescent kidney transplant recipients after kidney transplantation.

Research method: Qualitative research Semi-structured interviews

Study population: 22 adolescent kidney transplant recipients

Research themes: access to a sense of normalcy, facilitators, inhibitors, information needs

Data extraction：In October, 2024

1. Achieving a sense of normalcy

They identified a major theme of achieving a sense of normalcy. Being recognised as having the same opportunities and potential as their peers. Achieving a sense of normalcy contributes to better functioning, social adjustment, happiness and positive development after transplant. Those who were unable to achieve a sense of normalcy appeared to have difficulty adapting and coping with life after transplant. Five facilitators (Table 2) and five barriers (Table 3) to achieving a sense of normalcy were identified. ‘There aren't so many different things going on here that we're able to do what everyone else is doing.’ ‘It's been so long since the transplant, just starting a normal life and getting back into a routine. (Boy, 18) ‘When I didn't have a kidney transplant before I thought I was different, after the transplant I felt I was the same as everyone else’

2.Facilitating factors

(1) Being able to develop their own identity and access to acceptance and understanding from their peers, incorporating medication into their daily lives, feeling free and energised and having sufficient support structures and coping mechanisms to enable participants to achieve a sense of normality.

Developing their own identity:Receiving a kidney transplant gave participants a sense of stability in their lives, and most felt that they had been given a new opportunity for self-discovery, developing their personality and values. ‘I'm so much more confident now.’ I'm not very tall, everyone is much taller than me and I feel very shy. (Girl, 13)

Their self-esteem and confidence in their abilities and realising their potential has increased. It gives you more confidence to think you're not wrong.’ (Boy, 17)

Participants developed positive attitudes and set academic and career goals. Plan to study for a business degree next year and hope to work for an accountancy firm.’ (Boy, 17) / ‘I want to go to university for film studies.’ (Girl, 17)

They perceived themselves as healthy, and younger participants in particular felt that they could develop and grow normally.

(2) Peer Acceptance:For most, the kidney transplant enabled participants to attend school and participate in social activities. Being able to establish and maintain friendships with classmates or co-workers was important to participants. Having understanding, supportive and caring friends can enhance participants' self-confidence, positive coping behaviours and social adjustment. ‘I know who my friends are, the ones who are there for me through thick and thin.’ (Boy, 17) ‘My friends at school, I'm close to them.’ (Girl, 17) ‘I have great friendships with a lot of people who already know about the organ transplant I'm going to get.’ (girl, 17) ‘They know why I left school so they are nice and I don't have any problems with friends. (Boy, 14) ‘I think I'm normal, I have friends like everyone else.’ (Boy, 17 years old)

(3) Making medication routine:Most older adolescents accepted responsibility for medication and learned how to be vigilant when taking medication and incorporate it into their daily schedules. Over time, this becomes an effortless habit. Their understanding of the importance of preventing transplant rejection motivated them to take the medication. ‘Now it's starting to get into my brain.’ (Girl, 17 years old) ‘I i've been taking it, it's normal.’ (Girl, 17) ‘It's important to stick to your medication even if they give you side effects.’ I think the kidney rejection is worse than the side effects. (Girl, 18) ‘To prolong the life of my kidneys, I had to take medication to avoid having another kidney transplant.’ (Boy, 14) I'm good at planning things in advance so I'm always ready the night before or after I've had my medication. I have this form, this box, that I fill out once a week for everyone. (Boy, age 17)

（4) Freedom and energy:After the transplant, all participants were not subject to dietary and water restrictions. They realised that this increased their energy compared to living on dialysis or with chronic kidney disease, which made it possible for them to participate in school, sports and social activities. The sense of freedom and energy after transplantation was particularly noticeable in participants who had previously received dialysis. Kidney transplants alleviated feelings of test tubes, needles, long and frequent hospital visits, and constant sickness. ‘I can eat whatever I want, it's the best thing.’ (Girl, 17) ‘I feel normal again.’ I can do things that normal kids can do. When I was sick, I didn't know what it was like to live a normal life. How people can wake up in the morning without a headache, without feeling nauseous, without sleepiness. (Boy, 17) ‘I can do more things like fitness and sports so you can keep going all day without feeling tired.’ (Boy, 14 years old) ‘(Normal) is being able to play sports.’ (Boy, 16) ‘It's amazing.’ Together I never get up in the morning because I always feel nauseous, vomiting and sick. And now I can stand up and feel good. (Girl, 18) Before dialysis treatment I used to sleep in class but now I can have energy all day.’ (Girl, 17 years old) ‘I am allowed to do more things compared to before dialysis.’ (Boy, 12) ‘Well, I don't have to take injections morning and night anymore.’

(5) Support structure:Adolescents of all ages valued the support they received from their parents, who provided comfort, reassurance, and encouragement, and they appreciated the opportunity to connect with other transplant patients through formal activities such as kidney camps, transplant games, or informally during visits to the hospital. The comradery of shared experiences allowed participants to openly discuss their feelings, struggles, experiences, and coping strategies with each other. By talking with other adolescent transplant recipients, participants were better prepared psychologically and emotionally for what they would encounter before, during, and after their transplant. ‘My parents always told me, don't worry, it's not your fault, and that was reassuring. My parents' influence definitely played a major role. I felt bad at first, but they made me feel good. (Boy, 18) ‘I knew what was going to happen because dad had told me.’ For example, what would happen after the transplant and how long I would have to stay in hospital. What helped me the most was going to kidney camp and meeting other people.’ These people have been through what IÕd been through. It was easy to talk to anyone. (Girl, 17 years old) ‘You hear what they are going through, what they have been through and see how they are coping.’ You can talk to younger kids and sometimes help them understand things and maybe explain it from your point of view instead of the doctor's point of view and they might understand it better. (Girl, 17) ‘Even though the doctors have explained everything, it's a bit of an unknown, so it's more scary. But when you meet someone who's been through it makes you worry a bit less.' Older people who have had transplants help you understand what you're going to go through, not just what a transplant is.' (Girl, 17)

3. Obstructive factors

(1) Identity crisis:Some adolescents felt that their personalities and temperaments had changed after the transplant. They talked about losing self-esteem, feeling alienated and lonely, and becoming more withdrawn and isolated during the experience. They struggled to get on with good peers and lost confidence. Some were also concerned about physical underdevelopment. ‘I don't really know my personality now, I used to be shy but now I'm very outspoken. It's frustrating because I find it annoying not knowing what I'm going to be like at some point. A lot of people think it's a bit weird and it's hard to adapt. (Girl, 17) ‘My face is swollen and I've gained a lot of weight.’ (Girl, 14) ‘I gain weight when I'm on medication and it's something I hate.’ (Girl, 18) It's been 2 years since my transplant and I still haven't grown any taller.

(2) Peer Rejection:Adolescents face rejection and bullying from school peers. They can be teased for changes in their appearance, such as sudden weight gain (which is more of a problem in girls), excessive body hair, and a puffy face. This is particularly problematic in the first year after transplantation, when the side effects of immunosuppressive therapy are more pronounced. Some feel that their peers have abandoned them because they can't accept their new personality. This can lower their self-esteem and make them feel isolated, lonely and distrustful of others. For some, this problem was resolved over time. ‘I remember being called a coyote in fourth grade.’ It made it harder for me to trust people because I was always surrounded by the whole bullying. Getting over that, it's still something I find hard, but I do try to take it out of the equation. (Boy, 17) Like at school when people make fun of me I used to walk away and slam a wall.’ I withdrew a lot, I was scared to make friends because I was scared of getting hurt. (Girl, 17)’ ’I got made fun of in year 5 because I was too fat.’ (Girl, 13)

(3) Aversion to medication:Participants sometimes forgot to take their medication when they were concentrating on their studies or social activities. Some children still needed reminders from their parents. Some participants expressed frustration with the medication regimen as an unrelenting disruption to their lives, and they wanted to stop taking the medication for a while. Two participants mentioned that they felt ‘normal’ but that taking medication made them ‘different’ from others. One participant felt anxious and panicked when test results showed signs of being bad and took the medication for the exact time prescribed, but relaxed when the test results returned to normal. ‘Sometimes it took me a long time to shoot to be able to finish the task‘’ ‘just because of taking a different medication than everyone else.’ (Girl, 17) ‘My face is swollen and I've gained a lot of weight.’ I'm not happy. I'm sad that it's happening and I don't want to take the medication. (Girl, 14) ‘Sometimes I forget them and my mum has to keep reminding me.’ (Girl, 13) My blood tests showed that I was starting to show signs of rejection ...... My doctor told me to start taking the tablets every 12 hours.’ (When everything is normal), like I can do whatever I want now. (Girl, 17 years old)

(4) Lifestyle restrictions:Participants were advised by their doctors not to play sports with physical contact and were disappointed that they could not play sports such as football. Follow-up hospital tests, particularly in the first year after transplantation, and medication regimes meant that they felt restricted from making travel plans and making spontaneous decisions to spend nights away from home. ‘The side effects of being hairy caused a lot of teasing.’ (Boy, 14) I'm always worried that if I fall it will affect my kidneys.’ (Girl, 13) ‘I can't play rugby league.’ (Girl, 13) ‘I might be more careful about doing things, like running riot at school.’ (Boy, 16) ‘I can't do things I want to do, like play football.’ The doctor said no rough sports were allowed. (Boy, 12) ‘I have a blood test on Monday so I can only stay until Sunday.’ So it's a restriction, I can't stay outside. (Girl, 17) ‘I take a lot of medication, but it's not hard.’ It's a pain in the arse sometimes. You can't just suddenly decide to sleep in your friend's house because you won't have your medication with you. (Girl, 17)

(5) Fear and Uncertainty:Participants expressed fear of dialysis and uncertainty about the survival of their grafts, but for most, these reflections were inherently fleeting. Some participants said they realised that their kidneys would not last a lifetime, while others feared having to have another transplant or going back on dialysis. ‘I'm nervous and scared because I'm going to have to have dialysis treatment again. (Girl, 13) ‘I had a bad rejection at the beginning.’ I was nervous and scared because I was still in the hospital and bleeding every day. (Girl, 17) ‘I was a bit worried because it takes a long time for the kidney to recover.’ (Boy, 18) ‘A couple of years ago my blood tests showed signs of rejection and that was really worrying.’ (Girl 17)

4. Information needs

Participants mentioned that important information was to prepare other patients their age for what would happen during and after transplant. Most felt that they personally received enough medical information from their doctors. However, participants felt that doctors and other transplant recipients should impart a broader range of technical knowledge, medical and experiential aspects of transplantation so that younger patients are better prepared and have a sense of security about what they are going through. ‘They need to know how much not only the people around you, but you yourself, your appearance, has really changed.’ (Girl, 17) When you wake up, Tell Them All (a booklet) will connect with you. (Boy, 16) [A booklet] so that the kids know it's safe and they know what's going on because that's my main concern, those unknown things. (Boy, 14) Two older participants said that information about alcohol, drugs and substance use would be important, especially for older adolescents. ‘Doctors and nurses talk about everything but they mention drugs and alcohol very little. They need to talk more about the effects of alcohol. I don't drink a lot of alcohol. I drink small amounts. But what if I get older and drink heavily? How would that affect you? If they suddenly start drinking, they can really damage (the kidneys). However, if you know your limits beforehand, then maybe they won't go over the limit. (Girl, 17)There are also drugs, which are are illegal to take them, but thousands of people do. What happens if I decide to do it? (Girl, 17)
