# Supplementary material for: Life experiences after kidney transplantation in adolescents: A qualitative meta-synthesis
Source: PLoS One. 2025 Apr 9;20(4):e0321588. doi: 10.1371/journal.pone.0321588 (PMC11981148; doi:10.1371/journal.pone.0321588)
Supplement: S3 File — (DOCX) [file pone.0321588.s003.docx]

| **Examples of Search Strategy** | | |
| --- | --- | --- |
| **PubMed** | | |
| #1 | ((((((((((kidney transplantation[MeSH Terms]) OR (kidney transplantation[Title/Abstract])) OR (renal transplantation[Title/Abstract])) OR (renal transplantations[Title/Abstract])) OR (transplantations, renal [Title/Abstract])) OR (transplantation, renal[Title/Abstract])) OR (grafting, kidney[Title/Abstract])) OR (kidney grafting[Title/Abstract])) OR (kidney grafting[Title/Abstract])) OR (kidney transplantations[Title/Abstract])) OR (transplantations, kidney[Title/Abstract]) | 121055 |
| #2 | (((((((((((((adolescent[MeSH Terms]) OR (child[MeSH Terms])) OR (adolescent[Title/Abstract])) OR (adolescents[Title/Abstract])) OR (adolescence[Title/Abstract])) OR (teens[Title/Abstract])) OR (teen[Title/Abstract])) OR (teenager[Title/Abstract])) OR (teenagers[Title/Abstract])) OR (youth[Title/Abstract])) OR (youths[Title/Abstract])) OR (juvenile[Title/Abstract])) OR (students[Title/Abstract])) OR (children[Title/Abstract]) | 4138515 |
| #3 | ((((((((experience*[Title/Abstract]) OR (feeling*[Title/Abstract])) OR (perception*[Title/Abstract])) OR (view*[Title/Abstract])) OR (opinion*[Title/Abstract])) OR (perspective[Title/Abstract])) OR (psycho*[Title/Abstract])) OR (attitude*[Title/Abstract])) OR (thought*[Title/Abstract]) | 3659972 |
| #4 | (((((((((qualitative research[MeSH Terms]) OR (qualitative research[Title/Abstract])) OR (ethnographic[Title/Abstract])) OR (phenomenological[Title/Abstract])) OR (grounded[Title/Abstract])) OR (hermeneutics[Title/Abstract])) OR (descriptive[Title/Abstract])) OR (focus group[Title/Abstract])) OR (interview[Title/Abstract])) OR (content analysis[Title/Abstract]) | 578546 |
| #5 | #1 AND #2 AND #3 AND #4 | 23 |
| **Embase** | | |
| #1 | 'kidney transplantation':ab,ti OR 'renal transplantation':ab,ti OR 'renal transplantations':ab,ti OR 'transplantations, renal':ab,ti OR 'transplantation, renal':ab,ti OR 'grafting, kidney ':ab,ti OR 'kidney grafting':ab,ti OR 'transplantation, kidney ':ab,ti OR 'kidney transplantations':ab,ti OR 'transplantations, kidney':ab,ti | 89040 |
| #2 | 'adolescent':ab,ti OR 'child':ab,ti OR 'adolescents':ab,ti OR 'adolescence':ab,ti OR 'teens':ab,ti OR 'teen':ab,ti OR 'teenager':ab,ti OR 'teenagers':ab,ti OR 'youth':ab,ti OR 'youths':ab,ti 'juvenile':ab,ti OR 'students':ab,ti OR 'children':ab,ti | 2156256 |
| #3 | 'experience*':ab,ti OR 'feeling*':ab,ti OR 'perception*':ab,ti OR 'view*':ab,ti OR 'opinion*':ab,ti OR 'perspective':ab,ti OR 'psycho*':ab,ti OR 'attitude*':ab,ti OR 'thought*':ab,ti | 4823414 |
| #4 | 'qualitative research':ab,ti OR 'ethnographic':ab,ti OR 'phenomenological':ab,ti OR 'grounded':ab,ti OR 'hermeneutics ':ab,ti OR 'descriptive':ab,ti OR 'focus group':ab,ti OR 'interview ':ab,ti OR 'content analysis':ab,ti | 722603 |
| #5 | #1 AND #2 AND #3 AND #4 | 26 |
| **Web of Science** | | |
| #1 | (TS=(kidney transplantation) OR AB=(kidney transplantation OR renal transplantation OR renal transplantations OR transplantations, renal OR transplantation, renal OR grafting, kidney OR kidney grafting OR transplantation, kidney OR kidney transplantations OR transplantations, kidney)) | 78597 |
| #2 | (TS=(adolescent OR child) OR AB=(adolescent OR adolescents OR adolescence OR teens OR teen OR teenager OR teenagers OR youth OR youths OR juvenile OR students OR children)) | 2335056 |
| #3 | AB=(experience*OR feeling*OR perception*OR view*OR opinion* OR perspective OR psycho* OR attitude* OR thought*) | 4050518 |
| #4 | (TS=(qualitative research) OR AB=(qualitative research OR ethnographic OR phenomenological OR grounded OR hermeneutics OR descriptive OR focus group OR interview OR content analysis)) | 2180933 |
| #5 | #1 AND #2 AND #3 AND #4 | 19 |
| **Cochrane** | | |
| #1 | MeSH descriptor: [Kidney Transplantation] explode all trees | 4713 |
| #2 | (kidney transplantation OR renal transplantation OR renal transplantations OR transplantations, renal OR transplantation, renal OR grafting, kidney OR kidney grafting OR transplantation, kidney OR kidney transplantations OR transplantations, kidney):ti,ab,kw | 16884 |
| #3 | MeSH descriptor: [Adolescent] explode all trees | 137774 |
| #4 | MeSH descriptor: [Child] explode all trees | 82499 |
| #5 | (adolescent OR adolescents OR adolescence OR teens OR teen OR teenager OR teenagers OR youth OR youths OR juvenile OR students OR children):ti,ab,kw | 345449 |
| #6 | (experience*OR feeling*OR perception*OR view*OR opinion* OR perspective OR psycho* OR attitude* OR thought*):ti,ab,kw | 239251 |
| #7 | MeSH descriptor: [Qualitative Research] explode all trees | 2345 |
| #8 | (qualitative research OR ethnographic OR phenomenological OR grounded OR hermeneutics OR descriptive OR focus group OR interview OR content analysis):ti,ab,kw | 158435 |
| #9 | #1 AND #2 | 4713 |
| #10 | #3 AND #4 AND #5 | 39115 |
| #11 | #7AND #8 | 2345 |
| #12 | #9 AND #10 AND #11 | 5 |
| **CNKI** | | |
| #1 | TKA=(kidney transplantation OR renal transplantation OR renal transplantations OR transplantations, renal OR transplantation, renal OR grafting, kidney OR kidney grafting OR transplantation, kidney OR kidney transplantations OR transplantations, kidney) | 25548 |
| #2 | TKA=(adolescent OR adolescents OR adolescence OR teens OR teen OR teenager OR teenagers OR youth OR youths OR juvenile OR students OR children) | 155399 |
| #3 | TKA=(experience* OR feeling* OR view* OR perception* OR opinion* OR persceptive* OR psycho* OR attitude* OR thought*) | 130 |
| #4 | TKA=(qualitative research OR ethnographic OR phenomenological OR grounded OR hermeneutics OR descriptive OR focus group OR interview OR content analysis) | 11 |
| #5 | #1 AND #2 AND #3 AND #4 | 10 |
| **WANGFANG DATA** | | |
| #1 | TKA=(kidney transplantation OR renal transplantation OR renal transplantations OR transplantations, renal OR transplantation, renal OR grafting, kidney OR kidney grafting OR transplantation, kidney OR kidney transplantations OR transplantations, kidney) | 19817 |
| #2 | TKA=(adolescent OR adolescents OR adolescence OR teens OR teen OR teenager OR teenagers OR youth OR youths OR juvenile OR students OR children) | 710984 |
| #3 | TKA=(experience* OR feeling* OR view* OR perception* OR opinion* OR persceptive* OR psycho* OR attitude* OR thought*) | 32871 |
| #4 | TKA=(qualitative research OR ethnographic OR phenomenological OR grounded OR hermeneutics OR descriptive OR focus group OR interview OR content analysis) | 346154 |
| #5 | #1 AND #2 AND #3 AND #4 | 10 |
| **VIP** | | |
| #1 | TKA=(kidney transplantation OR renal transplantation OR renal transplantations OR transplantations, renal OR transplantation, renal OR grafting, kidney OR kidney grafting OR transplantation, kidney OR kidney transplantations OR transplantations, kidney) | 20498 |
| #2 | TKA=(adolescent OR adolescents OR adolescence OR teens OR teen OR teenager OR teenagers OR youth OR youths OR juvenile OR students OR children) | 674684c |
| #3 | TKA=(experience* OR feeling* OR view* OR perception* OR opinion* OR persceptive* OR psycho* OR attitude* OR thought*) | 1032634 |
| #4 | TKA=(qualitative research OR ethnographic OR phenomenological OR grounded OR hermeneutics OR descriptive OR focus group OR interview OR content analysis) | 11857 |
| #5 | #1 AND #2 AND #3 AND #4 | 1 |
| **EBSCO** | | |
| #1 | SU=kidney transplantation AND AB=( kidney transplantation OR renal transplantation OR renal transplantations OR transplantations, renal OR transplantation, renal OR grafting, kidney OR kidney grafting OR transplantation, kidney OR kidney transplantations OR transplantations, kidney ) | 31005 |
| #2 | SU=( adolescent or child ) AND AB=( adolescent OR adolescents OR adolescence OR teens OR teen OR teenager OR teenagers OR youth OR youths OR juvenile OR students OR children ) | 2452432 |
| #3 | AB=(experience*OR feeling*OR perception*OR view*OR opinion* OR perspective OR psycho* OR attitude* OR thought*) | 5036291 |
| #4 | SU= qualitative research AND AB=( qualitative research OR ethnographic OR phenomenological OR grounded OR hermeneutics OR descriptive OR focus group OR interview OR content analysis ) | 186478 |
| #5 | #1 AND #2 AND #3 AND #4 | 37 |
